# Effect of periprosthetic greater trochanter fractures (Vancouver AG) on primary stability of the femoral stem in total hip arthroplasty: a biomechanical micromotion analysis

**DOI:** 10.1007/s00068-026-03308-z

**Published:** 2026-08-24

**Authors:** Jan Bruder, Manuel Goronzi, Adrian Cavalcanti Kußmaul, Jan Wulf, Florian Pachmann, Boris Michael Holzapfel, Wolfgang Böcker, Manuel Kistler

**Affiliations:** Department of Orthopaedics and Trauma Surgery, Musculoskeletal University Center Munich (MUM), University Hospital, LMU Munich, Marchioninistr. 15, 81377 Munich, Germany

**Keywords:** Total hip arthroplasty, Periprosthetic fracture, Biomechanics, Micromotion

## Abstract

**Purpose:**

Primary stability is a crucial factor for the long-term success of cementless total hip arthroplasty (THA). Periprosthetic fractures represent an important cause of loss of primary implant stability. While Vancouver AG fractures rarely lead to stem loosening, their effect on the metaphyseal anchoring of the stems has not yet been investigated. The present study aimed to evaluate the influence of a Vancouver AG fracture on the primary stability of the CLS Spotorno stem.

**Methods:**

Six fourth-generation composite femora were implanted with cementless CLS Spotorno stems (Zimmer, Warsaw, USA). Primary stability was determined by three-dimensional digital image correlation under dynamic loading conditions (300–1700 N, 1 Hz) simulating physiological gait. Micromotion was recorded at six standardized measurement points (proximal, mid, distal, medial and ventral) before and after a simulated Vancouver AG fracture. Data was statistically analyzed using paired one-sided t-tests (α = 0.05).

**Results:**

The results revealed no significant increase in micromotion at the primary anchorage zone of the stem. However, a non-significant increase in micromotion was observed at the prosthesis tip (DGventral and DGmedial), where the micromotion values increased from 164.4 μm (± 59.6 μm) and 140.4 μm (± 75.6 μm) to 193.1 μm (± 48.8 μm) and 186.9 μm (± 60.5 μm), respectively.

**Conclusion:**

Vancouver AG fractures did not significantly affect the primary anchorage zone of the CLS Spotorno stem in this study. Further studies with larger sample sizes and varying implant designs are needed to confirm these findings and their clinical implications.

## Introduction

Total hip arthroplasty (THA) is the standard treatment for osteoarthritis of the hip and femoral neck fractures (FNF) and is performed with an increasing trend based on the current demographic changes. In the USA for example, it is expected that by 2030 more than 500.000 THAs will be performed annually. The implant durability is also increasing: After ten years, over 90% and after 20 years at least 80% of the THAs are still intact [1–4].

A decisive factor influencing the durability of the prosthesis is its primary stability [2]. In addition to aseptic loosening, periprosthetic fractures can also lead to loosening of the prosthesis’ stem and thus to a failure of the implant [3]. Data from national arthroplasty registers — including the Endoprothesenregister Deutschland (EPRD) and analyses from the American Joint Replacement Registry (AJRR) — as well as register-based cohort studies show that periprosthetic fractures, together with aseptic loosening, consistently rank among the most common reasons for revision after uncemented primary total hip arthroplasty [5–7].

In both the intraoperative and postoperative setting, approximately 24% of the periprosthetic femoral fractures involve the greater trochanter and can be classified as Vancouver AG fractures [8].

Generally, this fracture type is considered stable and predominantly treated conservatively. Yet, if the displacement of the trochanteric fragment is greater than 20 mm, open reduction and internal fixation (ORIF) is generally recommended due to the risk of gluteal insufficiency [9, 10]. Loosening of the prosthesis’ stem in periprosthetic AG fractures however is rare [11].

Recently, the stems of THAs are becoming shorter while their anchoring site is shifting proximally into the metaphyseal region. Currently, no study has yet investigated the effect of a Vancouver AG fracture on the micromobility of a metaphyseal anchored THA, which is why the aim of this biomechanical investigation is to determine the effect of a Vancouver AG fracture on the primary stability of the stem in the CLS Spotorno system. Results of a recent study have shown very high survival rates of 91.2% (or 95.1% if infections are excluded) after 18 years for the CLS Spotorno stem [12].

The authors hypothesized that the Vancouver AG fracture causes increased micromotion of the implanted stem and might result in a shortened durability of the prosthesis [12–15].

## Materials and methods

### Specimens and implants

For this study, the CLS Spotorno (CLS Spotorno, Zimmer, Warsaw, USA) size 13.75, Angle 135°, offset 44 mm was used. It is a straight stem prosthesis that is implanted cementless and has been in use since 1984 [13]. Planning before implantation of the stem was done via MediCAD 2D Hip (mediCAD Hectec GmbH, Altdorf/Landshut, Germany) planning software.

The prosthesis was implanted in six artificial femurs (large left 4th generation composite femur, #3406, Sawbones ^®^ Pacific Research Laboratories, USA) [14, 15].

A sample size calculation was performed a priori using the program G*Power (version 3.1.9.7; Heinrich Heine University Düsseldorf, Düsseldorf, Germany). Due to the standardized nature of the artificial bones and thus a generally lower variability compared to biological specimens, an effect size of dz = 1.19 was assumed to be appropriate. Based on an alpha level of 0.05 and a statistical power of 80%, the calculated sample size was six paired specimens.

### Specimen preparation

According to the official surgical technique, the femoral neck resection was performed 9 mm above the lesser trochanter. The resection angle was determined using a goniometer and a template.

The canal for the prosthesis was then gradually rasped. Starting with a size five rasp, a progressively larger rasp was used until adequate press fit stability was achieved and the canal matched the planned size. The shoulders of the implanted stems were positioned at a distance of 20 mm from the tip of the greater trochanter as described in the manufacturer’s manual.

In order to check the position of the implants, anterior-posterior and lateral radiographs were taken. The focus was on checking whether the stem was positioned centrally and straight in the femoral shaft.

**Fig. 1:**
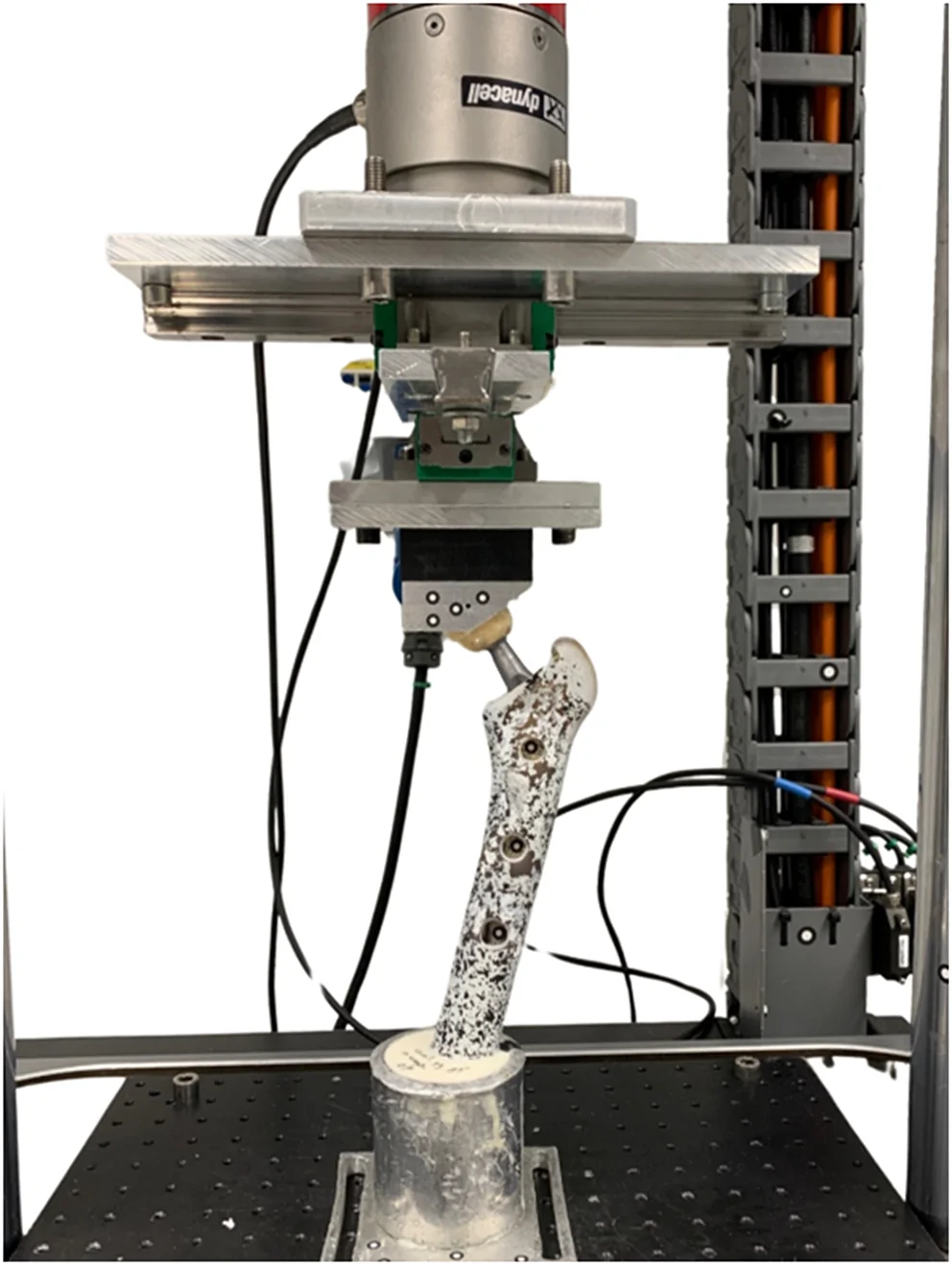
Experimental Setup for initial micromotion registration with intact major trochanter

The artificial bone was then sawed off 210 mm below the trochanter minor and embedded in a metal cup using polyurethane resin (Rencast FC 53, Gössl-Pfaff GmbH, Karlskron, Germany). According to in vivo data, the artificial bones were positioned as physiologically as possible. The specimens had an adduction angle of 16° in the frontal plane and a flexion angle of 9° in the sagittal plane [16, 17]. The following tests were then performed by using a standard 32 mm ceramic head of medium length (Fig. 1) [18–21].

**Fig. 2:**
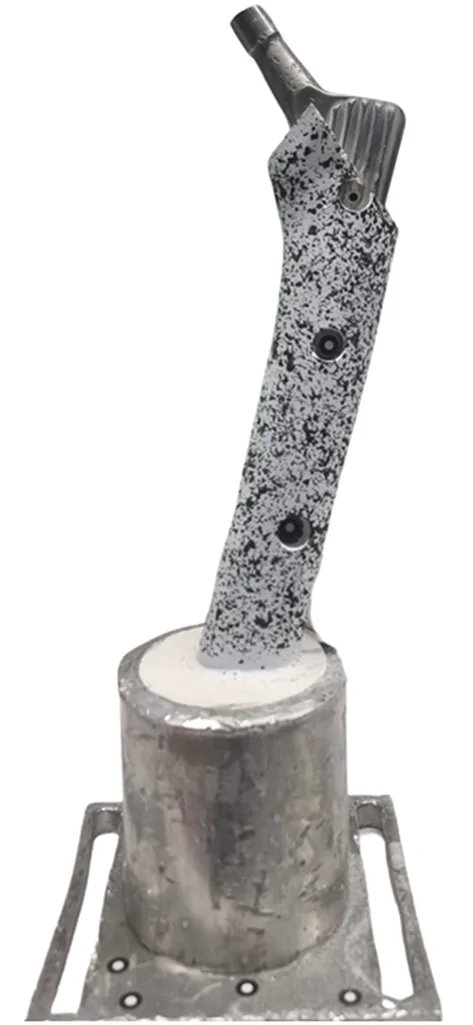
Resection of the greater trochanter

After the stems were implanted, the initial micromotion tests were conducted on all bones (micromotion procedure see below). Subsequently, the trochanter major was generously excised in all specimens. This involved a cut along the trajectory of the prosthesis neck. The resection exposed the posterior part of the CLS Spotorno prosthesis (Fig. 2).

### Micromotion registration

#### Loading procedure

To assess the degree of primary stability, all specimens underwent a standardized loading protocol utilizing sinusoidal dynamic loading patterns. The loading regimen, emulating the forces experienced during level walking, entailed dynamic loading ranging from 300 N to 1700 N at a frequency of 1 Hz. This was facilitated by a universal material testing machine (ElectroPuls E10000, Instron, Norwood, MA, U.S.). This loading scheme, aimed at mimicking the physiological stresses akin to a person weighing approximately 70 kg and was corroborated by previous in vivo measurements [16, 17]. Prior to data collection, a preconditioning phase consisting of 600 loading cycles ensured stability and mitigated the likelihood of prosthesis subsidence during subsequent measurements. The actual measurement at the six measurement points was performed simultaneously for the duration of 100 additional cycles. The loading was uniformly applied via a ceramic-on-ceramic joint affixed to an X-Y linear guideway, thus circumventing the introduction of shear forces.

#### Measurement of primary stability

**Fig. 3:**
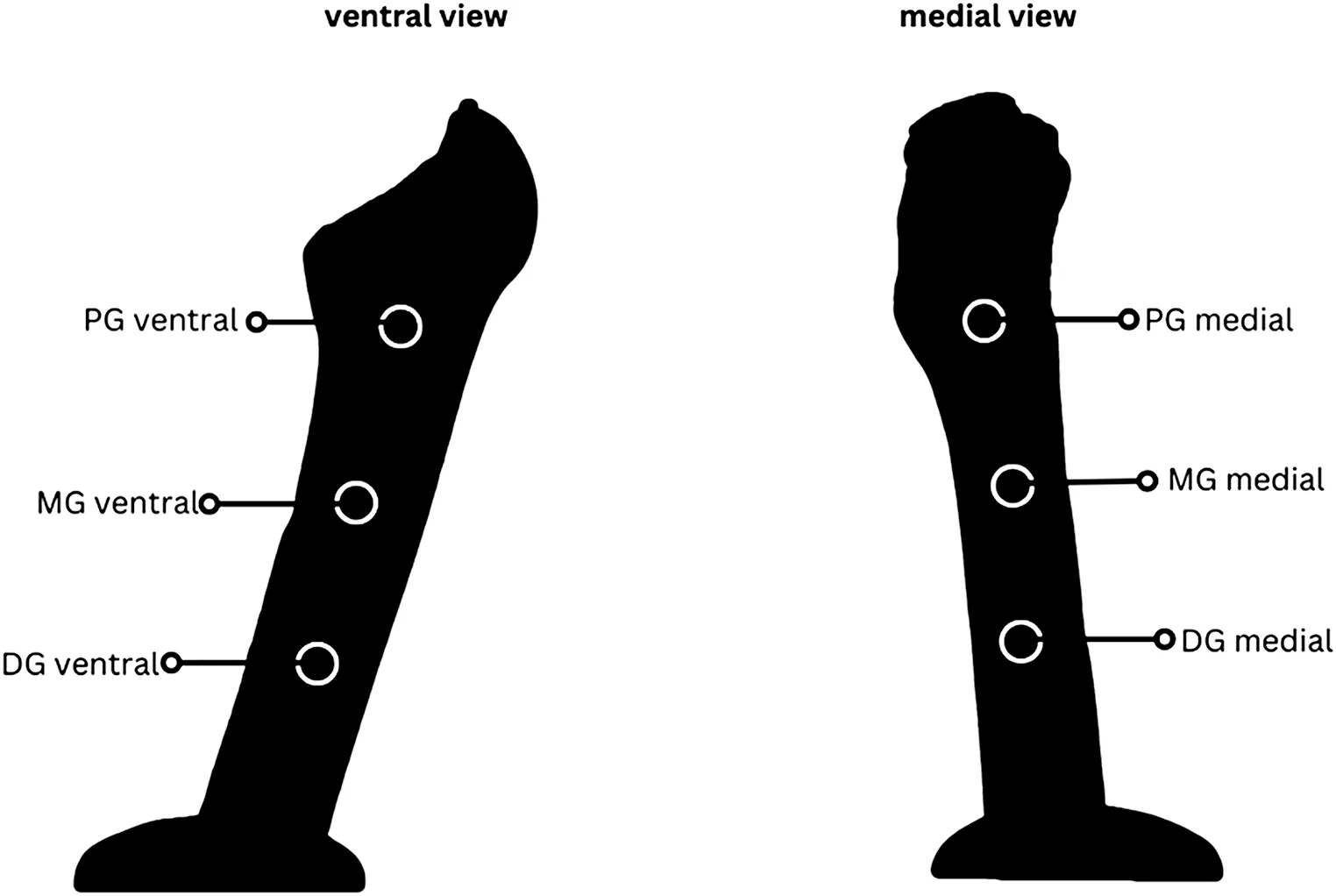
Illustration of the ventral (left) and medial (right) bone measurement points

Primary stability, denoting the magnitude of micromotion at the bone-implant interface, was assessed using three-dimensional (3D) digital image correlation. Micromotions were captured across six designated measuring positions, comprising three on the medial and three on the ventral side. The data were collected for PGventral (ventral proximal), PGmedial (medial proximal), MGventral (ventral mid), MGmedial (medial mid), DGventral (ventral distal), and DGmedial (medial distal) regions (Fig. 3). Proximal measurement points were situated at the level of the lesser trochanter, while distal points were positioned 15 mm above the stem tip. Midpoints were centrally located between proximal and distal points to ensure comprehensive data acquisition. An optical system, consisting of two cameras boasting a resolution of 1936 × 1216 pixels (ARAMIS 3D Camera 2.3 M, Carl Zeiss GOM Metrology GmbH, Braunschweig, Germany), facilitated measurements within a volumetric space of 560 mm x 380 mm x 380 mm, with a measuring distance of approximately 700 mm. The system exhibited an approximate measuring accuracy of 11.2 μm in the focus-plane and 22.4 μm out of the focus-plane, while strain measurements were accurate to within approximately 0.1%. To facilitate tracking of prosthesis movement, 10 mm diameter holes were drilled into the bone at designated measuring positions, and marker points were affixed directly onto the stem. A stochastic pattern, achieved by applying a thin layer of white paint overlaid with a speckle pattern of black paint (Aqua Eco+, European Aerosols GmbH, Hassmersheim Germany), was sprayed onto the bone surface. Following preconditioning, data acquisition was conducted at a rate of 5 Hz. The setup for this study is shown above (Fig. 1).

#### Data analysis and statistics

The relative 3D micromotion was determined by calculating the difference in movement between the bone and the hip stem at the six measurement positions for each cycle. To ensure accuracy, the motion of the bone was averaged from four measurement points within the stochastic pattern around each test position. This process was carried out separately for each coordinate axis (x, y, and z) before being combined into a 3D micromotion value. For statistical analysis the program R (Version 4.3.2, R Core Team, Vienna, Austria) was used. Firstly, the Shapiro-Wilk test was performed to assess whether the differences in micromotion values between intact and fractured greater trochanter at all measurement points were normally distributed. Then, a one-sided t-test for dependent variables was conducted to determine whether there was a significant difference in micromotion between the two conditions, based on a significance level of *p* < 0.05. The one-sided test was chosen based on the a priori directional hypothesis that the fracture would lead to increased micromotion, as a decrease in micromotion following an AG fracture was considered clinically irrelevant. The results of the Shapiro-Wilk test indicated that the differences between paired values were normally distributed (*p* > 0.05) for all variables beside one variable (MGmedial), which deviated from normal distribution (*p* < 0.05). However, since the t-test is considered robust against minor violations of the normality assumption, the analysis was still conducted using a paired t-test.

## Results

**Fig. 4:**
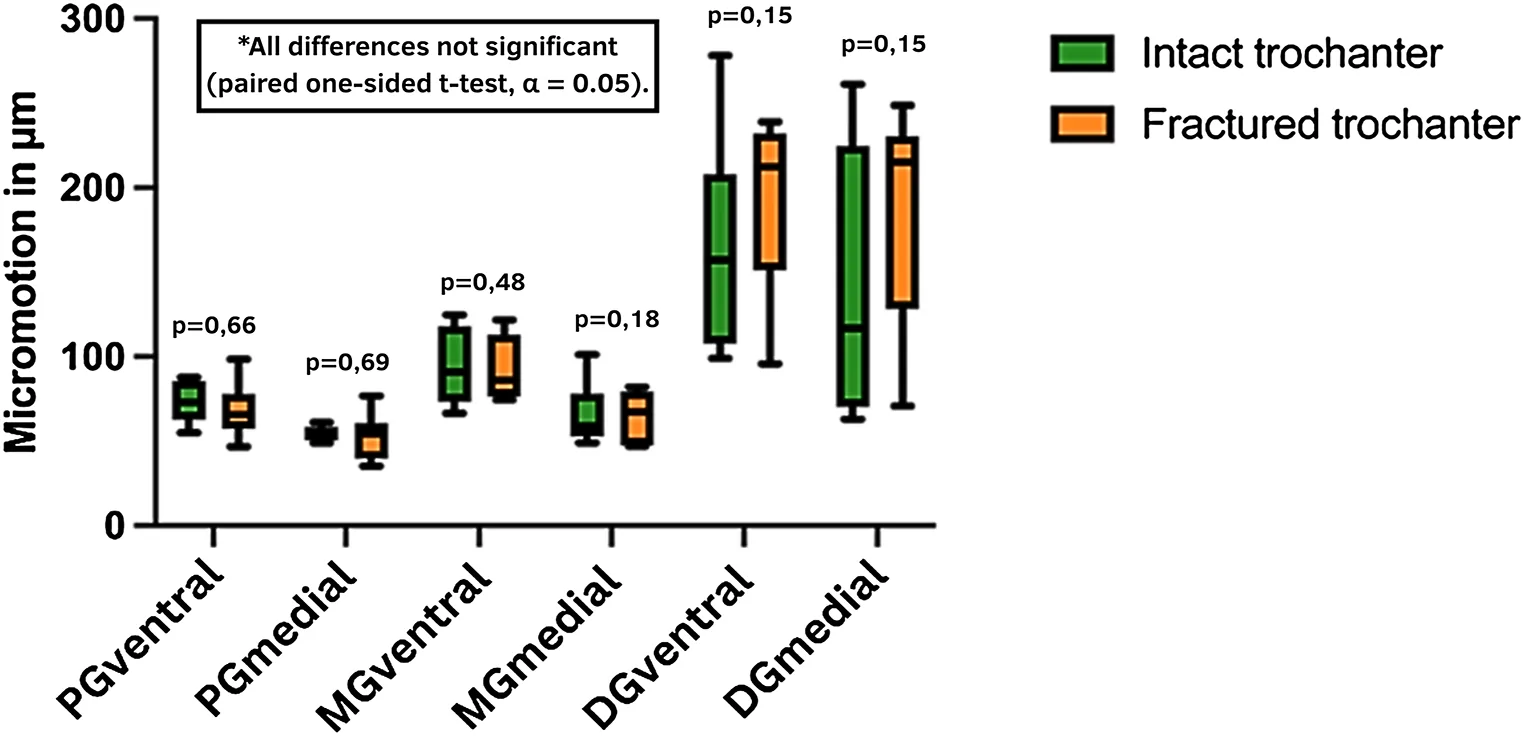
Primary stability (micromotion) before and after Vancouver AG fracture

The relative displacement between the stem and the surrounding bone (Micromotion) are presented in Table 1; Fig. 4.

**Table 1:** Micromotion [µm] before and after Vancouver AG fracture (mean (± SD))

	PGventral	PGmedial	MGventral	MGmedial	DGventral	DGmedial
Intact trochanter	73.1 (±11.7)	54.2 (±4.1)	94.0 (±20.8)	65.4 (±17.3)	164.4 (±59.6)	140.4(±75.6)
Fractured trochanter	67.9 (± 15.6)	52.8 (± 13.1)	92.4 (± 17.7)	64.8 (± 13.7)	193.1 (± 48.8)	186.9 (± 60.5)

### Proximal points

In the PGventral region, the micromotion for the intact trochanter was 73.1 μm (± 11.7 μm), while for the fractured trochanter it was 67.9 μm (± 15.6 μm). The PGmedial region showed a micromotion of 54.2 μm (± 4.1 μm) for the intact trochanter and 52.8 μm (± 13.1 μm) for the fractured trochanter.

### Mid points

The MGventral region recorded a micromotion of 94.0 μm (± 20.8 μm) for the intact trochanter, compared to 92.4 μm (± 17.7 μm) for the fractured trochanter. Similarly, in the MGmedial region, the micromotion was 65.4 μm (± 17.3 μm) for the intact trochanter and 64.8 μm (± 13.7 μm) for the fractured trochanter.

### Distal points

In the DGventral region, the micromotion increased from 164.4 μm (± 59.6 μm) for the intact trochanter to 193.1 μm (± 48.8 μm) for the fractured trochanter. The DGmedial region showed an increase in micromotion from 140.4 μm (± 75.6 μm) in the intact trochanter to 186.9 μm (± 60.5 μm) in the fractured trochanter.

Overall, the results revealed that the presence of a fractured trochanter tends to increase micromotion in most regions, particularly in the DGventral and DGmedial regions. The differences in micromotion were less pronounced in the PG and MG regions.

The highest micromotion, and also the highest difference between intact and fractured, was observed at the two distal points (DGventral and DGmedial) at the tip of the prosthesis stem. However, based on a one-sided t-test for dependent variables, no statistical significance in micromotion was observed in all points.

## Discussion

The interpretation of the micromotion values measured in the present study requires reference to established biomechanical thresholds. A widely cited upper limit of 150 μm has traditionally been used to demarcate the boundary above which fibrous tissue ingrowth rather than osseointegration is expected. In the present study, micromotion at the distal measurement sites already exceeded this value under intact conditions (DGventral: 164.4 μm; DGmedial: 140.4 μm), and increased further following simulated AG fracture (DGventral: 193.1 μm; DGmedial: 186.9 μm). However, this threshold must be interpreted with caution in the context of the CLS Spotorno stem, which features a smooth, non-hydroxyapatite-coated tip specifically designed to preclude osseointegration at the stem tip and thereby prevent stress shielding in the proximal anchorage zone. A systematic review and meta-analysis by Kohli et al. underscores this point, demonstrating that the 150 μm threshold is not universally applicable but is strongly modulated by implant surface characteristics, including the presence of hydroxyapatite coating, pore geometry, and loading intervals [22]. Their analysis of 51 studies found that successfully osseointegrated implants exhibited a mean micromotion of 112 ± 176 μm, whereas osseointegration failure was associated with a mean of 349 ± 231 μm. Importantly, the failure threshold reported by Kohli et al. carries a standard deviation of ± 231 μm, resulting in a wide distribution ranging from approximately 118 μm to 580 μm. The post-fracture distal micromotion values recorded in the present study (DGventral: 193.1 μm; DGmedial: 186.9 μm) thus fall well within this range, and cannot be classified as clearly safe relative to the failure boundary. This observation underscores that a potentially negative long-term effect on implant stability cannot be definitively excluded on the basis of the present data alone.

The clinical relevance of this biomechanical observation is further supported by the study of Oetojo et al., who reported outcomes of 18 patients with minimally displaced or non-displaced Vancouver AG fractures managed non-operatively [23]. Radiological union was achieved at a mean of 14.2 weeks, pain resolved in all but one patient, and no cases of periprosthetic stem loosening were documented during follow-up. The absence of stem loosening in this clinical series is consistent with the present study’s finding that the AG fracture did not significantly compromise micromotion in the proximal metaphyseal anchorage zone of the CLS Spotorno stem (PGventral: 73.1 vs. 67.9 μm; PGmedial: 54.2 vs. 52.8 μm), where primary and secondary implant stability are predominantly achieved. Taken together, the data from Kohli et al. and Oetojo et al. suggest that the increase in distal micromotion observed following AG fracture in the present study may carry clinical relevance that warrants serious consideration: the post-fracture values fall within the distribution associated with osseointegration failure identified by Kohli et al., and the clinical series by Oetojo et al., limited to non-displaced fractures managed conservatively, cannot be directly extrapolated to the biomechanical conditions modelled here. It must be acknowledged, that the observed micromotion increases did not reach statistical significance in the present study, which is likely attributable to the limited sample size. This absence of statistical significance does not preclude clinical relevance, but rather underscores the necessity of further studies with larger sample sizes to determine whether the observed trends are reproducible and, if so, whether they translate into measurable clinical consequences. Future investigations should additionally examine varying prosthesis designs to assess whether stems with different anchoring mechanisms or surface characteristics are more susceptible to the effects of an AG fracture.

Our study focused on the impact of a Vancouver type AG fracture on the primary stability of the cementless implant CLS Spotorno, commonly used in total hip arthroplasty (THA). The main findings were the increased micromotion at the two distal points (DGventral and DGmedial) comparing the non-fractured and the fractured model, whereas no differences were detected at the other measurement sites. Indeed, considering the primarily metaphyseal anchorage provided by the CLS Spotorno stem [13, 24], the trochanteric fragment did not appear to have a marked negative effect on this anchorage.

Our findings confirm that micromotion is more pronounced at the distal points of the prosthesis, as previously demonstrated by other studies [13, 18–24]. This phenomenon can be attributed to the design of the CLS Spotorno, which facilitates proximal load transfer. The slender and narrow tip of the prosthesis is not intended for osseous integration, aiming to prevent excessive stress shielding in the proximal region and thus reducing bone mineral density (BMD) [13, 25]. It is important to note that to avoid osseous integration, the micromotion at the tip should not fall below a value of 150 μm [26]. On the other hand, too high micromotion should also be avoided since too low primary stability may result in a higher risk of prosthesis long-term failure [27], hence it is necessary to find the right balance of all such factors for best performance and longevity.

We have seen an increase in the micromotion values around the tip of the prosthesis with the presence of the fracture, although these were not statistically significant. Micromotion values without a fracture were 164.4 μm (± 59.6) at DGventral and 140.4 μm (± 75.6) DGmedial. These values increased to 193.1 μm (± 48.8) at DGventral and 186.9 μm (± 60.5) DGmedial in the presence of a fracture. Therefore, our findings suggest that a fractured trochanter may compromise the primary stability of the prosthesis, potentially impacting the longevity of the total hip arthroplasty.

Although the observed differences did not reach statistical significance, it cannot be excluded that an increase in micromotion at the stem tip may develop clinical relevance over time, potentially contributing to the progression of aseptic loosening and, subsequently, to increased micromotion at other sites. Due to the limitations of our study, including the small sample size, the clinical relevance of these findings cannot be confirmed. Rather, the presented data should be regarded as hypothesis-generating and as a basis for further research. It should furthermore be noted that, beyond prosthesis stability, the hip abductors attach to the greater trochanter; therefore, displacement of the trochanteric fragment could contribute to abductor insufficiency and functional instability of the THA joint. This represents an additional clinical rationale for fixation of displaced greater trochanter fragments, independent of the findings of the present study.

The CLS Spotorno is one of the early metaphyseal anchored stems. At the time of its market launch in 1984, a paradigm shift took place. In order to reduce stress shielding in the stem area, metaphyseal anchored prostheses were developed to replace the diaphyseal anchored designs that had been used until then. In comparison to contemporary prostheses that feature shorter stems and exclusively metaphyseal anchoring, the CLS Spotorno prosthesis can be regarded as a stem with “hybrid anchoring,” exhibiting metaphyseal-orientation with diaphyseal support. The influence of a periprosthetic AG fracture on a purely metaphyseal anchoring stem could be greater, as there is even less diaphyseal support to be expected [12, 13, 25].

In addition, only synthetic bone models and a single prosthesis design were employed in the experimental setup. However, this particular prosthesis represents a well-established design whose biomechanical principles have served as the foundation for the development of several currently used implant models.

However, the use of standardized biomechanical bone models allows excellent reproducibility and comparability. Due to the lack of anatomic variation and different bone densities it can be assumed that the forces applied within the loading protocol were transmitted to every specimen in the same way. Further studies with cadaver models should be performed to confirm the findings of this study.

Furthermore, several methodological aspects should be explicitly acknowledged as limitations. In the present study, the greater trochanter was completely excised rather than fractured, and no soft-tissue attachments were preserved. In a realistic clinical scenario, a Vancouver AG fracture retains partial muscle and capsular connections to the trochanteric fragment, which may influence load distribution around the prosthesis and differ from the conditions modelled here. Additionally, patients with a Vancouver AG fracture are typically managed with partial weight bearing in the postoperative period. The full physiological loading conditions applied in this study therefore represent a worst-case scenario and may not reflect the actual mechanical environment during clinical recovery. It should, however, be noted that in elderly patients, who represent the primary population affected by periprosthetic fractures, the ability to comply with partial weight-bearing restrictions is often severely limited. Kammerlander et al. demonstrated that the majority of older patients with hip fractures are unable to maintain prescribed postoperative weight-bearing restrictions [28]. In this context, the loading conditions applied in the present study may more accurately reflect the actual in vivo situation for this patient population than a partial weight-bearing protocol would suggest.

Despite these limitations, our findings represent an important addition to the current knowledge regarding the behavior of this prosthesis in the presence of AG fractures and outline the need for further research considering different implant designs and loading conditions.

## Conclusion

The findings of the present study indicate that a Vancouver AG fracture does not result in increased micromotion within the primary anchorage zone of the metaphyseal anchored Spotorno stem. Nevertheless, a non-significant increase in micromotion was detected at the prosthesis tip. Although not statistically significant, this observation warrants further investigation in studies with larger sample sizes and varied implant designs. The present data should be regarded as hypothesis-generating and as a basis for further research.

## Data Availability

All data supporting the findings of this study are available within the paper and its Supplementary Information.
